# Prevalence and clinical correlates of intimate partner violence (IPV) among women with mental illness

**DOI:** 10.1192/j.eurpsy.2025.427

**Published:** 2025-08-26

**Authors:** A. Jeyagurunathan, E. Abdin, M. I. M. K. Shah, Y. T. Lee, V. D. Sagayadevan, S. Shafie, R. Sambasivam, Z. Yunjue, K. Xinyi, L. M. F. Eng, T. C. C. Ting, S. Basu, N. Chandwani, S. Y. L. Lee, J. Liu, S. A. Chong, M. Subramaniam

**Affiliations:** 1 Research Division; 2 Department of Psychosis; 3 Department of Mood and Anxiety, Institute of Mental Health, Singapore, Singapore

## Abstract

**Introduction:**

Intimate partner violence (IPV) is a major public health concern. One of the most common forms of interpersonal violence concerns IPV, one in three women which is approximately 35% of women who experience physical and sexual violence by an intimate partner at some points in their lives. Women with mental illness are a vulnerable risk group for IPV.

**Objectives:**

The current study aimed to assess the prevalence and clinical correlates of IPV among women outpatients with mental illness in a tertiary care psychiatric hospital.

**Methods:**

118 participants with a primary diagnosis of schizophrenia spectrum disorders or depression were recruited. Data on intimate partner violence (IPV) were assessed on the World Health Organization Violence Against Women (WHOVAW) scale, consisting of three domains-psychological, physical and sexual intimate partner violence. Psychopathology was measured using Brief Psychiatric Rating Scale-18 items (BPRS) questionnaire, consisting of five domains- positive symptoms, negative symptoms, resistance symptoms, activation symptoms, and affect symptoms. Data on socio-demographic characteristics were also obtained. Multivariable logistic regression was used for analysis.

**Results:**

The mean (SD) age of women participants was 32.63 years (10.96). The overall prevalence of IPV among women with mental illness was 55.1%. Participants who were separated/widowed/divorced (versus single) were significantly more likely to experience total VAW scores (OR=14.57), and psychological (OR=21.64), and physical (OR=11.30) domains. Those who belong to Malay ethnicity (versus Chinese ethnicity) were significantly more likely to experience sexual abuse (OR=6.25). Women who were unemployed (versus employed) were significantly more likely to experience sexual IPV (OR=3.94). Women who experienced IPV (OR=1.36), psychological abuse (OR=1.30) and physical abuse (OR=1.25) were significantly more likely to have positive symptoms compared to those who did not experience IPV. Women who experienced IPV (OR=1.14) and psychological abuse (OR=1.13) were significantly more likely to have affect symptoms compared to those who did not experience IPV.

**Conclusions:**

The study highlights the prevalence of IPV among women with mental illness. Overall VAW scores, psychological and physical IPV were strongly associated with higher score on the positive and affect symptoms on psychopathology scale. The high prevalence of IPV among this group of patients is concerning and mental health professionals should actively identify IPV and implement holistic interventions to ensure good care of women with mental illness.

**Disclosure of Interest:**

None Declared

